# Rapid, Serial, Non-invasive Assessment of Drug Efficacy in Mice with Autoluminescent *Mycobacterium ulcerans* Infection

**DOI:** 10.1371/journal.pntd.0002598

**Published:** 2013-12-19

**Authors:** Tianyu Zhang, Si-Yang Li, Paul J. Converse, Jacques H. Grosset, Eric L. Nuermberger

**Affiliations:** 1 Center for Tuberculosis Research, Department of Medicine, Johns Hopkins University School of Medicine, Baltimore, Maryland, United States of America; 2 State Key Laboratory of Respiratory Diseases, Center for Infection and Immunity, Guangzhou Institutes of Biomedicine and Health, Chinese Academy of Sciences, Guangzhou, Guangdong, the People's Republic of China; 3 Department of International Health, Johns Hopkins Bloomberg School of Public Health, Baltimore, Maryland, United States of America; Universidad Peruana Cayetano Heredia, Peru

## Abstract

**Background:**

Buruli ulcer (BU) caused by *Mycobacterium ulcerans* is the world's third most common mycobacterial infection. There is no vaccine against BU and surgery is needed for patients with large ulcers. Although recent experience indicates combination chemotherapy with streptomycin and rifampin improves cure rates, the utility of this regimen is limited by the 2-month duration of therapy, potential toxicity and required parenteral administration of streptomycin, and drug-drug interactions caused by rifampin. Discovery and development of drugs for BU is greatly hampered by the slow growth rate of *M. ulcerans*, requiring up to 3 months of incubation on solid media to produce colonies. Surrogate markers for evaluating antimicrobial activity in real-time which can be measured serially and non-invasively in infected footpads of live mice would accelerate pre-clinical evaluation of new drugs to treat BU. Previously, we developed bioluminescent *M. ulcerans* strains, demonstrating proof of concept for measuring luminescence as a surrogate marker for viable *M. ulcerans in vitro* and *in vivo*. However, the requirement of exogenous substrate limited the utility of such strains, especially for *in vivo* experiments.

**Methodology/Principal Finding:**

For this study, we engineered *M. ulcerans* strains that express the entire *luxCDABE* operon and therefore are autoluminescent due to endogenous substrate production. The selected reporter strain displayed a growth rate and virulence similar to the wild-type parent strain and enabled rapid, real-time monitoring of *in vitro* and *in vivo* drug activity, including serial, non-invasive assessments in live mice, producing results which correlated closely with colony-forming unit (CFU) counts for a panel of drugs with various mechanisms of action.

**Conclusions/Significance:**

Our results indicate that autoluminescent reporter strains of *M. ulcerans* are exceptional tools for pre-clinical evaluation of new drugs to treat BU due to their potential to drastically reduce the time, effort, animals, compound, and costs required to evaluate drug activity.

## Author Summary

The discovery and development of new drugs to improve the treatment of BU is greatly hampered by the slow growth rate of the organism, which requires up to 3 months to form countable colonies on solid media. Here, we engineered a virulent reporter strain of *M. ulcerans* with intrinsic bioluminescence to enable serial, real-time, non-invasive *in vivo* monitoring of viable bacterial counts and demonstrate its utility for high-throughput *in vitro* and *in vivo* screening of antibiotic efficacy using a panel of anti-mycobacterial drugs with various mechanisms of action. We show: 1) that a drug's *in vitro* activity against *M. ulcerans* is detectable in real time after as little as 2 days of exposure, and 2) that a drug's *in vivo* activity against *M. ulcerans* is detectable in as little as 1 week through rapid, serial, non-invasive assessment in live mice performed with a benchtop luminometer. This method promises dramatic reductions in time and effort as well as requirements for animals, compounds and other supplies. We believe such a strain is capable of transforming drug discovery and development efforts for BU.

## Introduction

Buruli ulcer (BU) caused by *Mycobacterium ulcerans* is the world's third most common mycobacterial disease with cases occurring on every continent, especially in certain humid tropical regions of the world [1], [2]. *M. ulcerans* secretes an immunosuppressive macrolide toxin, termed mycolactone [3] whose biosynthetic enzymes are encoded on a giant plasmid [4]. Mycolactone is responsible for deep and necrotizing skin ulcers and occasionally bone lesions. No vaccine is available for BU [5]. Based on experiments in the mouse footpad model [6], [7] and subsequent clinical studies [8]–[12], a regimen of streptomycin (STR) plus rifampin (RIF) for 2 months is recommended for treatment of BU [13], although additional surgery including skin grafting may be necessary to repair large ulcers, contractures and deformities [10]. Though this 2-month drug regimen does reduce numbers of colony-forming units (CFU), lesion size, and mycolactone levels [14], [15], it has significant disadvantages, including STR's requirement for parenteral administration and potential for oto- and vestibulotoxicity, while RIF causes challenging drug-drug interactions with many drugs, including anti-mycobacterial and anti-retroviral agents. Therefore, entirely oral regimens and/or regimens capable of treating BU in 1 month or less are sought [16].

Efforts to evaluate the therapeutic potential of drugs for BU in the pre-clinical setting are hampered by the very slow growth of *M. ulcerans* which necessitates up to 3 months of incubation at 32°C for colonies to form on solid media. Light production by various luciferase enzymes has been used as a real-time biomarker of bacterial viability for high-throughput screening of antibiotics and drug susceptibility testing against mycobacteria [17]–[22]. The bacterial luciferases encoded by *luxAB* catalyze the oxidation of reduced flavin mononucleotide using a long-chain fatty aldehyde substrate, producing H_2_O and light (∼490 nm wavelength) in the process. Other genes of the lux operon (*luxCDE*) encode enzymes for the synthesis of the aldehyde substrate [23]. We have recently demonstrated that recombinant bioluminescent reporter strains of *M. ulcerans* expressing *luxAB* genes from *Vibrio harveyi* are useful for real-time evaluation of drug activity *in vitro* [21] and *in vivo* [22]. The endpoints used to measure efficacy in this mouse footpad model were relative light units (RLU) detected *ex vivo* in the footpad tissue or *in vivo* in live mice. However, these recombinant strains require the exogenous addition of substrate to produce light, making serial monitoring of the infection in live mice challenging. More recently, we created a virulent and stable autoluminescent *Mycobacterium tuberculosis* strain and demonstrated its utility for high-throughput *in vitro* and *in vivo* screening of antibiotic efficacy [24]. Using this reporter strain for serial, non-invasive monitoring of live mice in an acute infection model, drug activity against *M. tuberculosis* was evident within 3 days of treatment. In the present study, we created a virulent and stable autoluminescent *M. ulcerans* strain and evaluated its utility for real-time evaluation of antimicrobial effects *in vitro* and rapid, serial, non-invasive assessment of efficacy in two mouse footpad infection models.

## Materials and Methods

### Ethics statement

All animal procedures were conducted according to relevant national and international guidelines. The study was conducted adhering to the Johns Hopkins University guidelines for animal husbandry and was approved by the Johns Hopkins Animal Care and Use Committee, protocol MO08M240. The Johns Hopkins program is in compliance with the Animal Welfare Act regulations and Public Health Service (PHS) Policy and also maintains accreditation of its program by the private Association for the Assessment and Accreditation of Laboratory Animal Care (AAALAC) International.

### Antibiotics

Rifampin (RIF), streptomycin (STR) and isoniazid (INH) were purchased from Sigma (St. Louis, MO). Kanamycin (KAN) and hygromycin (HYG) were purchased from Invitrogen (Carlsbad, CA) and Roche Diagnostics (Indianapolis, IN), respectively. Moxifloxacin (MXF), linezolid (LZD), clarithromycin (CLR), and bedaquiline (BDQ, formerly known as TMC207), were kindly provided by Bayer (Leverkusen, Germany), Pfizer (New York, NY), Abbott (Abbott Park, IL), and Tibotec (Beerse, Belgium), respectively.

STR, RIF and MXF were dissolved in distilled water, and CLR and LZD were dissolved in distilled water with 0.05% agarose for administration to mice. BDQ was formulated in an acidified cyclodextrin suspension as previously described [25]. All drugs were administered 5 days per week in 0.2 ml. RIF, MXF, CLR and LZD were administered by esophageal gavage. STR was administered by subcutaneous injection. The daily dosages are indicated for each experiment.

### Creation and selection of autoluminescent *M. ulcerans* (AlMu) strains

We previously constructed plasmids for expressing the *luxCDABE* operon from *Photorhabdus luminescens* [26] in mycobacteria, including the episomal (pTYOEH) and integrative (pTYOK, pTYZOK1/pTYZOK2 and pOAIK1/pOAIK2) constructs [27]. pTYOK contains only one copy of *luxCDABE* from *P. luminescens* under control of one *hsp60* promoter. pTYZOK contains one copy of *luxAB* from *V. harveyi* and one copy of *luxCDABE* from *P. luminescens*, each under one *hsp60* promoter. pOAIK1 and pOAIK2 contain an additional copy of *luxB* from *V. harveyi* downstream of *luxCDABE* [27], with the only difference between plasmids being the orientation of the fragment inserted. We transformed these plasmids into colony suspensions of *M. ulcerans* Mu1059 (WtMu, a clinical isolate from Ghana [28]) by electroporation, as described previously [21]. Two months later, colonies isolated on KAN- or HYG- containing selective 7H11 plates were individually tested for luminescence. Positive transformants for each plasmid were picked, homogenized in 2 ml Dubos broth with 0.07% Tween 80, and incubated at 32°C. When the OD_600 nm_ reached more than 0.3, luminescence was detected using a TD-20/20 luminometer (Turner BioSystems), measuring light production over 3 sec. Strains were compared on the basis of relative light units (RLU) per ml of culture and the ratio of RLU to colony-forming units (CFU).

### Real-time *in vitro* drug susceptibility testing in liquid media

Serial dilutions of drug-containing solutions and *M. ulcerans* broth culture (OD600 of 0.3 to 0.7) were prepared as previously described [21]. RLU counts from the same batch of triplicate samples were measured daily over the first 7 days of exposure. The TD20/20 luminometer provides RLU values 1,000 times lower than those provided by the 20/20^n^ luminometer. For consistency with prior studies, we defined 1 RLU as 1 unit in the 20/20^n^ luminometer, equivalent to 0.001 units in the TD20/20 luminometer [21].

### *In vivo* growth curves of AlMu and WtMu

Colony suspensions of AlMu and WtMu were made by vortex-mixing 30 mg of colony material in 15 ml PBS. After allowing the clumps to settle, the resulting suspension was used to inject the right hind footpads of six-week old, female BALB/c mice. The inoculum volume was 0.03 ml, containing approximately 4 log_10_ CFU. The left hind footpads served as negative controls for observation of swelling. On the day after infection and at each time point thereafter, 5 mice per strain were sacrificed after non-invasive *in vivo* RLU measurement to determine RLU and CFU counts in the right hind footpad suspension. The mice were first anesthetized by isoflurane inhalation and the *in vivo* RLU count was determined non-invasively by placing the foot into the detection hole of the luminometer and measuring light production for 4 sec. To assure reproducibility, three separate measures were obtained for each mouse. The mice were then euthanized. The footpads were carefully cleaned with antimicrobial soap and water followed by an alcohol swab. After cleaning, the footpads were harvested using scalpel and forceps, minced in one drop of PBS using scissors and suspended in 1 ml PBS. After being shaken several times, the suspension was allowed to stand for 10 minutes to allow larger tissue debris to settle down. The resulting supernatant was used for *ex vivo* RLU detection, which was performed twice. Series of 100-fold dilutions of the suspension (undiluted, 10^−2^, 10^−4^) were plated in duplicate directly onto selective 7H11 plates (0.5 ml sample/plate).

### Time to footpad swelling and serial, non-invasive, real-time monitoring of drug activity in a murine model of *M. ulcerans* disease

Mice infected as described above were examined on a weekly basis to determine the footpad lesion index, which is defined as follows: 0 = normal footpad; 1 = non-inflammatory footpad swelling; 2 = inflammatory footpad swelling; 3 = inflammatory hindfoot swelling; 4 = inflammatory leg swelling; and 5 = death of the mouse [6]. For the purpose of assessing time-to-swelling, swelling was defined as a lesion index of grade 2 or higher. Treatment began 31 days after infection, when all footpads reached a swelling index ≥2 and continued for 3 or 4 weeks. At each time point, 5 mice from each group were sacrificed for RLU (*in vivo* and *ex vivo*) and CFU counts. Colonies of AlMu isolated from untreated control mice 8 weeks after infection were assessed for autoluminescence to confirm stability of the construct *in vivo*.

### Serial, non-invasive, real-time monitoring of drug activity in a murine model of preventive treatment

Mice were infected as described above. RLU counts determined non-invasively on the day after infection as described above were used to allocate mice to treatment groups (4 mice per group) with comparable distributions of RLU counts. Treatment began either 1 day or 11 days after infection and was administered for 2 weeks. The following 3 dose levels of each drug were used: 40, 10 and 2.5 mg/kg of body weight for RIF; 150, 75 and 19 mg/kg for STR; 100, 25 and 6.3 mg/kg for CLR; 25, 6.3 and 1.6 mg/kg for BDQ; and 200, 100 and 25 mg/kg for MXF. RLU counts from live mice were measured non-invasively in whole footpads of live mice twice weekly during treatment and on the footpad homogenate at the time of sacrifice, when CFU counts were also determined.

### Data analysis

RLU and CFU counts were log_10_ transformed before analysis. Group means were compared by unpaired *t* test or by one-way analysis of variance (ANOVA) with Dunnett's posttest when multiple comparisons were made. An alpha value of 0.05 was used to determine statistical significance. Time-to-swelling curves were compared using the log rank test. As 5 pairwise comparisons were made in the time-to-swelling analysis, an alpha value of 0.01 was used to determine statistical significance. All statistical tests were performed with Prism 4 software (GraphPad Software, Inc., San Diego, CA).

## Results

### Selection of autoluminescent *M. ulcerans* strains

**Figure 1:**
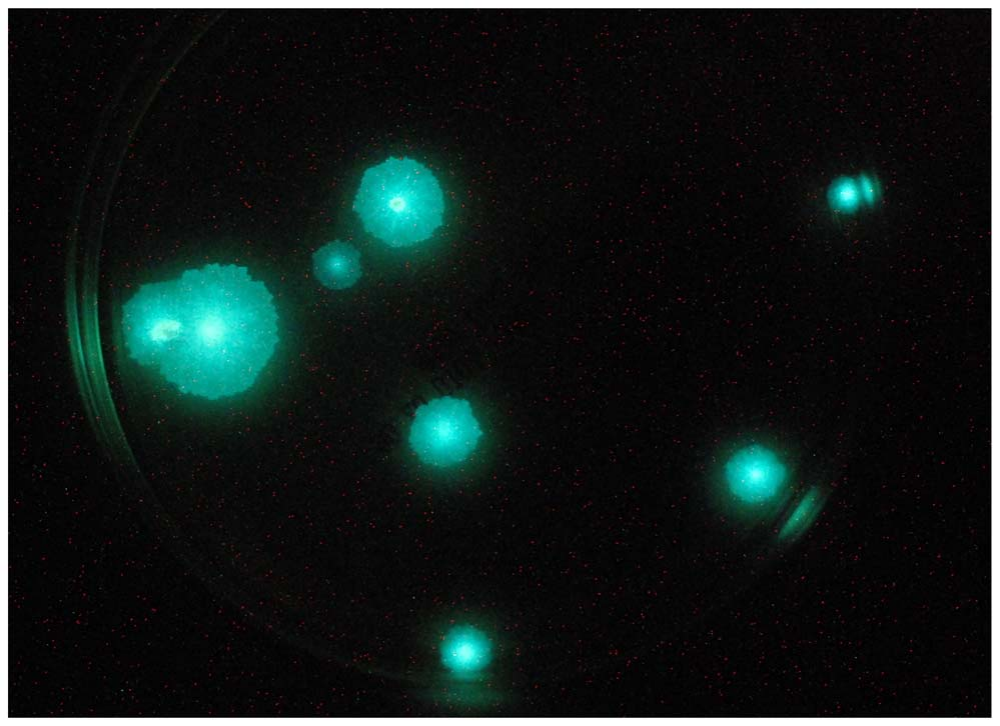
Photograph of autoluminescent *M. ulcerans* colonies taken with a one-minute exposure time.

The natural *luxCDABE* operon from *P. luminescens* [26] under control of the constitutive *hsp60* promoter [24] was successfully cloned into four different vectors. However, only colonies transformed with the integrative plasmids pTYOK and pTYZOK2 exhibited strong luminescence that was visible to the naked eye and captured by a digital camera (Figure 1). Similar results were observed when engineering an autoluminescent *M. tuberculosis* strain [27]. In addition, the colonies grew as fast as their parent strain on agar plates. In vivo, the doubling time for WtMu was calculated at 4.93 days (95% confidence interval, 3.5–8.3) and for AlMu it was 3.63 days (95% confidence interval, 2.4–7.6). Colonies transformed with the other vectors produced weaker or no light, implying that the orientation of the *luxCDABE* operon is important and that increasing expression of *luxAB* with additional copies does not increase light production. The strain containing pTYOK was selected for further study because it did not contain additional copies of *luxAB* in addition to the *luxCDABE* operon.

### Real-time *in vitro* drug susceptibility testing

**Figure 2:**
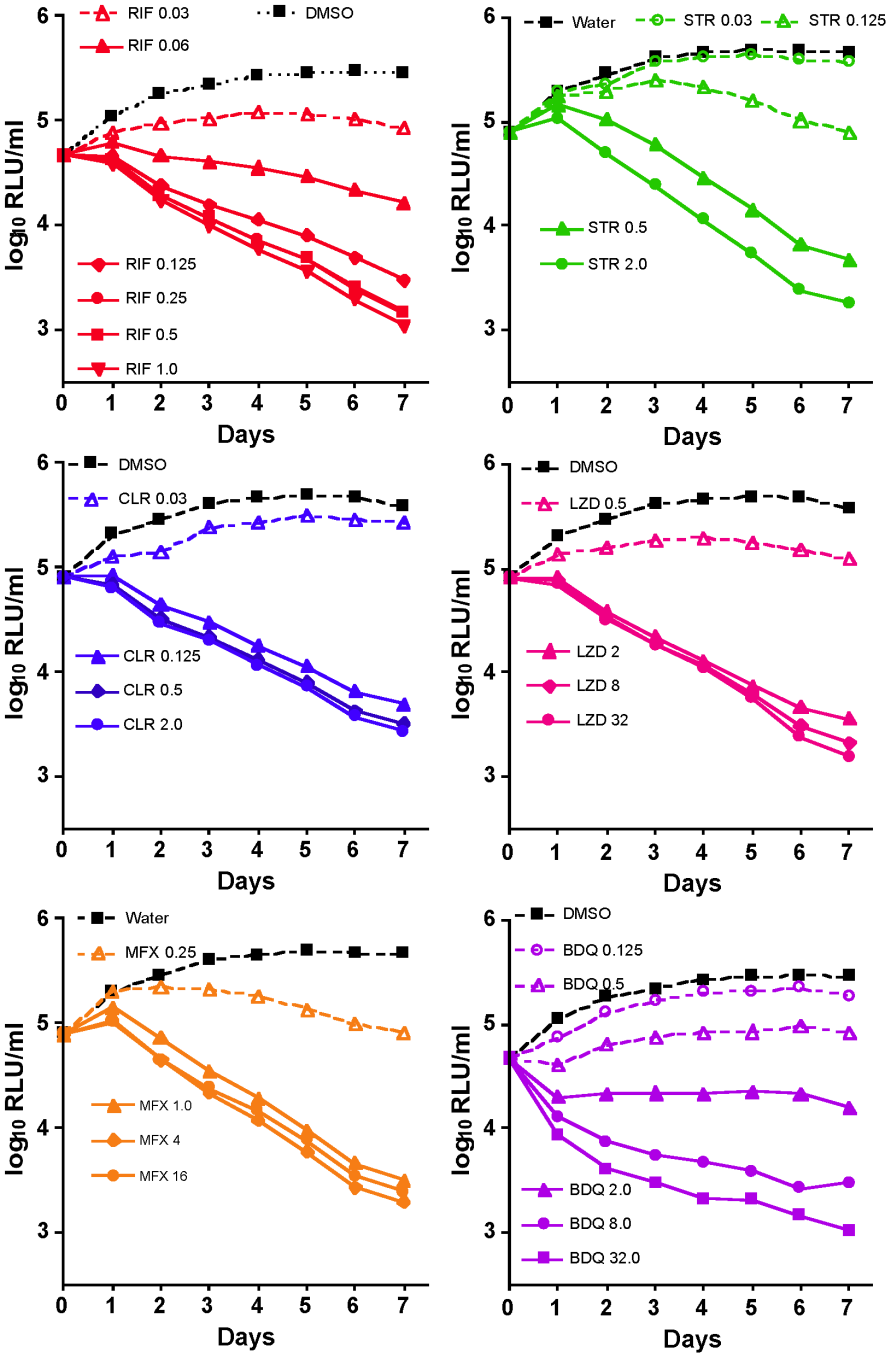
Results of real-time drug susceptibility testing *in vitro*. The solid line with solid triangle in each panel indicates the MIC_lux_ of each drug, as defined in the text. Concentrations below the MIC_lux_ of each drug are shown with open symbols and broken lines. Concentrations above the MIC_lux_ have solid symbols and lines. Untreated (diluent) controls are in black. The background luminescence level was 2.7 log_10_ RLU/ml.

Serial RLU counts were obtained daily, without adding exogenous substrate, from tubes of 7H9 broth containing AlMu in the presence of each of 6 anti-mycobacterial drugs with different mechanisms of action. The activity of each drug increased with concentration. BDQ had the greatest effect on RLU counts over the first 1–2 days, but the maximal effect observed over 7 days was similar for each drug. We defined the MIC_lux_ as the lowest concentration that prevented an increase in RLU after 7 days of incubation, i.e., RLU_day7_≤RLU_day0_. MIC_lux_ values against AlMu were similar to the MIC against the WtMu parent strain (Table 1) [21]. Even closer correspondence may have been achieved if doubling dilutions of drug had been used. Time-kill curves are shown in Figure 2. The MIC_lux_ could be discriminated from the drug-free control by the 2^nd^ day of incubation. In each case, RLU remained detectable above background after 7 days of drug exposure, demonstrating a suitable dynamic range to the assay.

**Table 1:** Comparison of MICs of selected antibiotics for autoluminescent *M. ulcerans* strain 1059 (AlMu) and its wild-type parent strain.

Drug	MICs (µg/ml) for WtMu on 7H11 Agar (previous study, [21])	MIC_lux_ (µg/ml) by AlMu for 7 days (this study)
RIF	0.13	0.06
STR	0.25	0.50
CLR	0.25	0.13
MXF	>0.50	1.0
LZD	1	2
INH	>40	>20
BDQ	Not tested	2

### Growth and virulence of AlMu and WtMu strains in the murine footpad model

**Figure 3:**
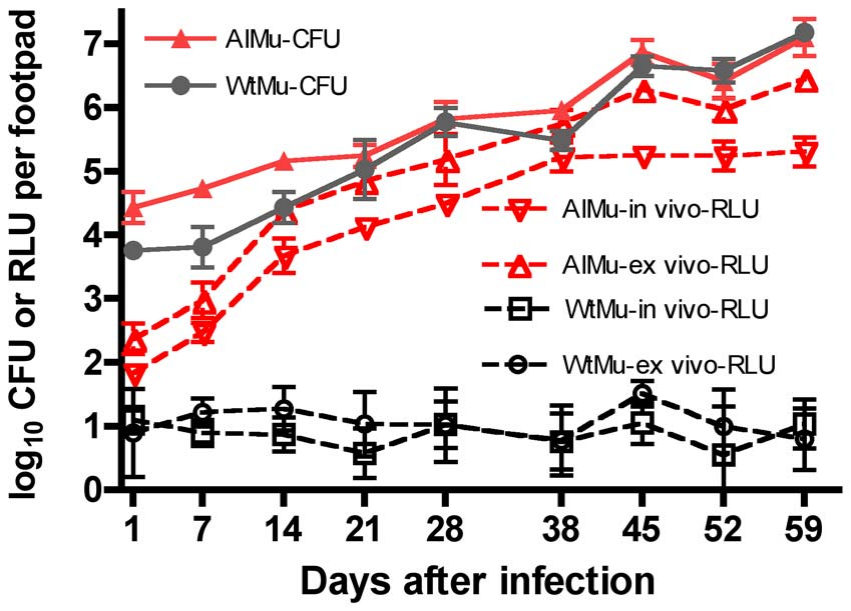
RLU and CFU counts over time following mouse footpad infection with AlMu or WtMu strains. The background luminescence level of this method is 1.7 log_10_ RLU/footpad. RLU-in vivo, light from live mice; RLU-ex vivo, light from footpad suspensions. Wild-type *M. ulcerans* (WtMu, black); autoluminescent *M. ulcerans* (AlMu, red). CFU results, solid lines and symbols; RLU results, broken lines and open symbols.

The mean RLU count of the AlMu suspension used to infect mice was 6.52 (SD 0.12) log_10_ RLU/ml. The light produced from footpads of live mice infected with AlMu increased continuously before reaching a plateau around 38 days post-infection, largely parallel to the change in RLU and CFU counts measured from the corresponding footpad suspension obtained at sacrifice (Figure 3). The RLU counts obtained non-invasively from live mice and those from footpad suspensions collected at necropsy in the AlMu groups were strongly correlated with the CFU counts, with correlation coefficients (r^2^) of 0.91 and 0.95, respectively. No significant light was detected from live mice or footpad suspensions from the WtMu infected group. The number of bacilli injected into the footpad was 3.76 (SD 0.11) for WtMu and 4.44 (SD 0.24) for AlMu. Swelling occurred 4–5 weeks post-infection for most mice (Figure S1). These results were comparable to a past experiment with similar infectious doses [22]. Thus, the AlMu strain appears to grow just as well and be just as virulent as the wild-type parent strain.

### Serial, non-invasive, real-time monitoring of drug activity in a murine model of *M. ulcerans* disease

**Figure 4:**
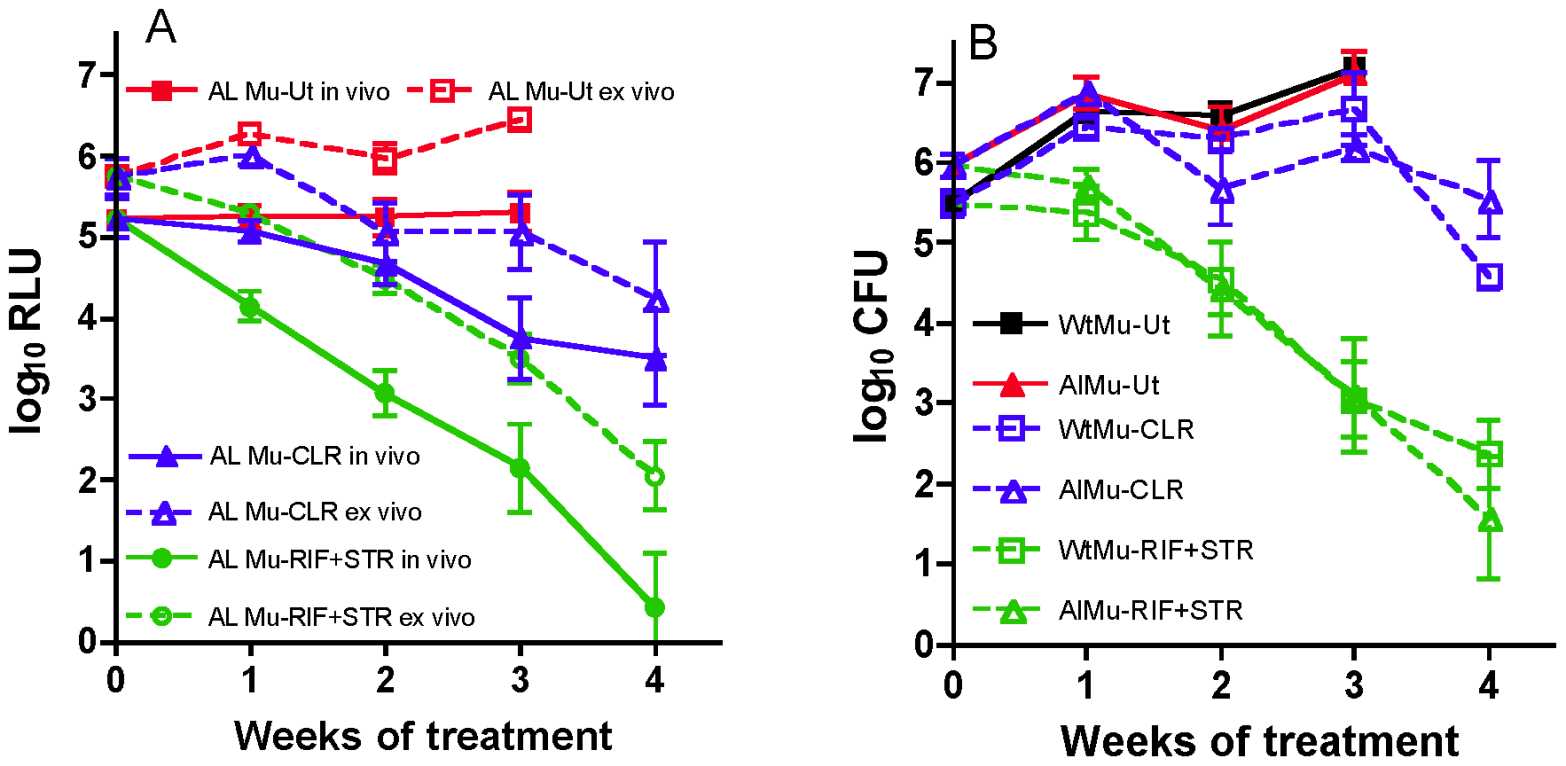
Results of footpad RLU (A) and CFU (B) counts obtained during treatment in the murine model of *M. ulcerans* disease. Beginning 31 days post-infection, mice with a footpad lesion index ≥2 received no treatment (Ut, red for autoluminescent strain, black for wild type), CLR alone (blue) or STR+RIF (green) for 4 weeks. RLU counts (A) were determined non-invasively from footpads of live autoluminescent (AlMu)-infected mice (*in vivo*, solid lines) or from footpad suspensions of the same mice after sacrifice (*ex vivo*, broken lines). CFU counts (B) were determined from the same footpad suspensions (autoluminescent, triangles) and from those of wild-type (WtMu, squares)-infected mice.

By day 31 after infection, the infected right hind footpads of all mice showed swelling. After treatment initiation with either STR+RIF or CLR alone, the impact of treatment on RLU counts could be observed within 2–4 days. Within one week of treatment, mice treated with STR+RIF experienced an approximately 1 log_10_ greater reduction in RLU counts compared to those receiving CLR alone (Figure 4). For AlMu-infected mice treated with STR+RIF or CLR alone, the effect on RLU counts measured non-invasively correlated well with the effects of treatment on RLU and CFU results determined *ex vivo* from footpad suspensions from the same animals, even though STR+RIF was highly bactericidal whereas CLR alone reduced the CFU counts by roughly 1 log_10_ CFU from the peak CFU count (Figure 4). For example, the correlation coefficients relating the non-invasive RLU counts to the *ex vivo* RLU and CFU counts were 0.99 and 0.98, respectively. Moreover, the responses of AlMu and WtMu to the regimens in this established disease model were very similar (Figure 4). We did not find any colony that lost the ability to produce light, suggesting that the AlMu construct is stable in animals even in the face of treatment with a highly bactericidal regimen.

The RLU counts from live mice treated with STR+RIF for only 3 weeks continued to decrease at week 4 and week 5 despite no treatment (Figure S2), which indicated a strong post antibiotic effect. In the case of CLR, the RLU counts remained largely stable for 2 weeks after treatment, suggesting the post-antibiotic effect was not as great. The mean log_10_ CFU count per footpad from AlMu-infected mice treated for 4 weeks with RIF+STR or CLR alone were 2.59 (SD 0.82) and 3.72 (SD 1.09), respectively. In support of the observed post-antibiotic effects, the mean CFU counts from AlMu-infected mice treated for 3 weeks with RIF+STR or CLR alone and sacrificed 2 weeks later were 2.11 (SD 0.53) and 5.25 (SD 0.32) (Figure S2).

### Serial, non-invasive, real-time monitoring of drug activity in a murine model of preventive treatment

**Figure 5:**
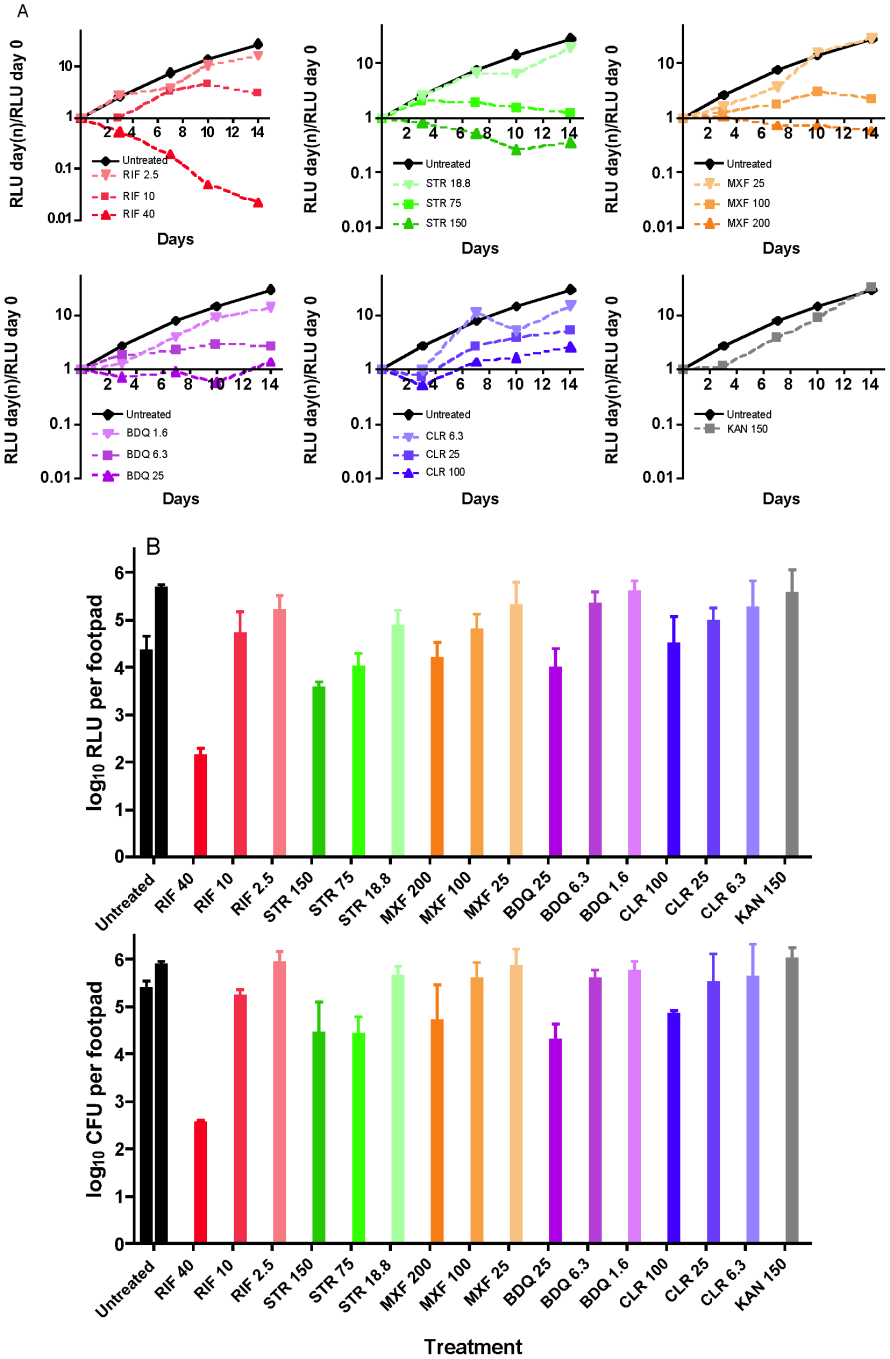
Correlation of footpad RLU and CFU counts during preventive drug treatment of AlMu-infected mice. Beginning 11 days post-infection, mice received monotherapy with different doses of RIF (red), STR (green), MXF (orange), CLR (blue) or BDQ (purple). Untreated (Ut, black) and KAN (gray)-treated mice served as negative controls. Serial RLU counts obtained non-invasively (A) are compared with RLU and CFU counts from footpad suspensions obtained from the same mice sacrificed after 2 weeks of treatment (B).

Using a preventive model of testing drug activity by initiating treatment 11 days after infection, before the onset of footpad swelling, we observed dose-dependent activity of drugs with differing mechanisms of action, as measured by RLU obtained non-invasively (Figure 5A). The AlMu strain has a KAN resistance marker; KAN was used as a negative control and showed no activity at 150 mg/kg. RIF and STR showed bactericidal activity at 40 and 150 mg/kg, respectively. However, RIF at 10 mg/kg and STR at 75 mg/kg displayed only bacteriostatic activity and no activity at lower doses. Similarly, MXF at 200 mg/kg and BDQ at 25 mg/kg only had bacteriostatic activity and weaker activity at lower doses. CLR at 100 mg/kg had bacteriostatic activity in this experiment but none at lower doses. Drug activities can be observed within one week of treatment initiation in live mice (Figure 5A). Similar results were obtained using RLU and CFU counts from footpad suspensions (Figure 5B).

**Figure 6:**
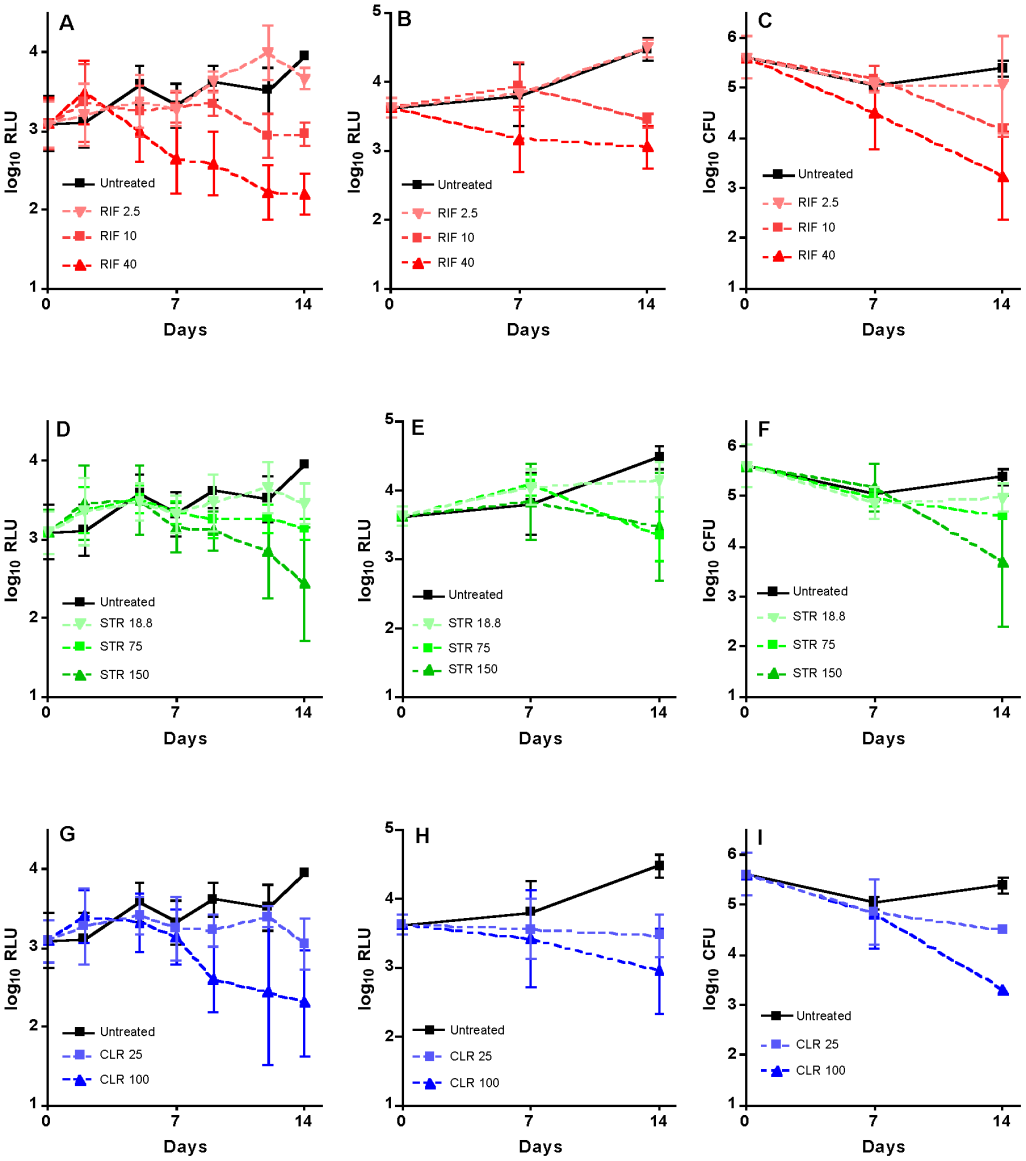
Footpad RLU and CFU values obtained from AlMu-infected mice treated from the day after infection. The mice were treated with RIF (red), STR (green) and CLR (blue) alone. Untreated (black). (A, D, G): RLU values obtained non-invasively from live mice (N = 8 on days 0–7; N = 4 on days 9–14); (B, E, H): RLU values obtained from footpad suspensions (N = 4 on days 7 and 14); (C, F, I): CFU values obtained from footpad suspensions (N = 4 on days 7 and 14). Values are mean ± SD.

We also attempted to examine drug activity more rapidly by initiating treatment the day after infection. Differentiation of bactericidal and bacteriostatic activity in this system could be assessed after 10–14 days, but not after 7 days, for STR and CLR, whereas high-dose RIF activity could be discerned in the first week whether by RLU from the footpads of live mice or by RLU and CFU from footpad suspensions (Figure 6).

## Discussion

*M. ulcerans* requires up to 3 months of incubation at 32°C to form colonies on solid media, hampering the pre-clinical evaluation of new drugs and drug regimens to improve BU treatment. We have engineered an autoluminescent *M. ulcerans* strain and shown the advantages of using it as a real-time surrogate marker for viable bacilli to overcome this impediment. We have improved upon our previous studies [21], [22] in this regard by developing a truly non-invasive method for more rapid (e.g., approximately 60 mice can be assessed per hour by one person) and simple detection of light emitted from viable *M. ulcerans* infecting mouse footpads without the requirement for exogenous administration of substrate and using serial, real-time *in vivo* monitoring of *M. ulcerans* infection to evaluate the response to treatment.

As reported previously with *M. tuberculosis* [27], creation of an autoluminescent *M. ulcerans* strain could not be achieved using an extra-chromosomal vector but was successful using an integrative plasmid, indicating that *M. tuberculosis* and *M. ulcerans* have similar regulatory mechanisms for producing light with *luxCDABE* and maintaining extra-chromosomal plasmids. Growth, virulence, and drug susceptibility (with the necessary exception of KAN due to the inserted resistance marker) of AlMu are equivalent to its parental strain.

Our *in vitro* test system, using only 200 µl of the AlMu strain in broth, can discern the bacteriostatic and bactericidal activity of a drug within 2–3 days of exposure, indicating its potential for high-throughput screening. It is possible to obtain the MIC_lux_ within 1 week and, through the generation of dynamic time-kill curves, it may be possible to obtain useful information on drug pharmacodynamics, such as concentration- or time-dependence [29]. None of the tested drugs was able to reduce RLU below the limit of detection (2.7 log_10_/ml) in 7 days, indicating a suitable dynamic range for the assay. In addition, as also shown with autoluminescent *M. tuberculosis* as well as recombinant *M. ulcerans* expressing *luxAB* alone, the RNA- and protein-dependent generation of light production is not affected by antibiotics inhibiting transcription or translation out of proportion to the effects on CFU counts. Interestingly, BDQ, which inhibits ATP synthesis, did reduce RLU counts more sharply than other drugs over the first 2 days of exposure. AlMu displayed the same drug susceptibility as its parent strain. Taken together, these results indicate that AlMu is a suitable reporter strain for testing drug activity *in vitro*. Although the construction and evaluation of only a single autoluminescent *M. ulcerans* strain is a clear limitation of the present study, the limited published data regarding *in vitro* drug susceptibility among *M. ulcerans* strains do not indicate marked strain-to-strain differences [30]–[32].

The AlMu strain showed the same growth rate, virulence, and drug susceptibility in mice as its parent strain, indicating its utility as a reporter strain for *in vivo* drug efficacy studies. In these studies, we determined that a drug could be shown to be inactive, bacteriostatic, or bactericidal as early as two weeks after infection and treatment initiation in a preventive model. Treatment time can be further reduced to one week if treatment initiation is delayed to two weeks after infection, allowing for the use of a smaller amount of compound. If there is activity in the preventive model, testing in the established disease model may more closely mimic clinical presentations with swollen tissue and inflammation. In this model, in which the bacterial burden is more-or-less stable in the absence of treatment, RLU obtained non-invasively from live mice indicated that the STR+RIF regimen is bactericidal but the impact of the bacteriostatic CLR monotherapy regimen was more difficult to discern. With the established disease model, one can also keep mice for an additional 2–3 weeks to see if treatment has succeeded in killing bacteria and preventing relapse. Finally, it is likely that this autoluminescent strain could also be helpful in evaluating vaccine efficacy by monitoring the same mice over time after challenge.

## Supporting Information

Figure S1 **Time to footpad swelling in mice infected with wild-type** ***M. ulcerans*** **(WtMu, black open symbol, broken line) and a recombinant luminescent** ***M. ulcerans*** **strain that required the addition of an exogenous substrate (rlMu, red open symbol, broken line) from a previous study compared to the same measure in mice infected with WtMu (black, solid symbol, solid line) or the autoluminescent strain (AlMu, red solid symbol, solid line) in the current study.** In both experiments, all strains induced swelling in most mice within 4 to 5 weeks of infection.
(TIF)

Figure S2 **Post antibiotic effects of clarithromycin and combined rifampin and streptomycin.** (A) Mice (N = 5 per group) with swollen footpads were treated for 3 weeks (broken lines) and had weekly RLU assessments for up to 5 weeks to determine the post antibiotic effects of clarithromycin (CLR, blue) monotherapy or rifampin and streptomycin (RIF+STR, red) combination therapy. Additional mice (N = 5 per group) were treated for 4 weeks and sacrificed at treatment completion (solid lines). (B) CFU counts in footpads of mice treated for 3 weeks and then assessed 2 weeks later (hatched bars) or treated for 4 weeks and then assessed at treatment completion (solid bars).
(TIF)
