# Supplementary figures and images for: Rapid, Serial, Non-invasive Assessment of Drug Efficacy in Mice with Autoluminescent Mycobacterium ulcerans Infection
Source: PLoS Negl Trop Dis. 2013 Dec 19;7(12):e2598. doi: 10.1371/journal.pntd.0002598 (PMC3868507; doi:10.1371/journal.pntd.0002598)

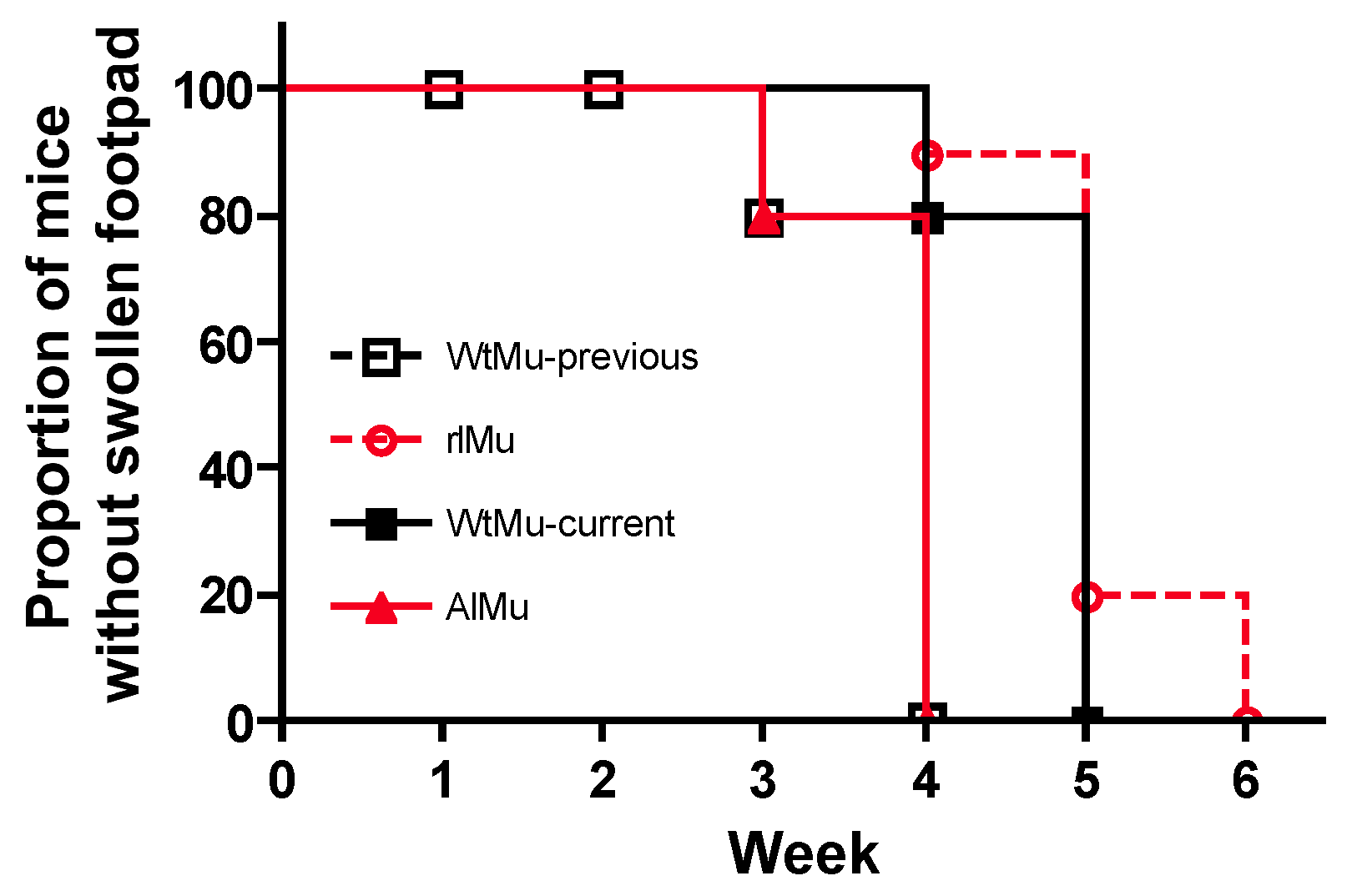

Supplement: Figure S1 — Time to footpad swelling in mice infected with wild-type M. ulcerans (WtMu, black open symbol, broken line) and a recombinant luminescent M. ulcerans strain that required the addition of an exogenous substrate (rlMu, red open symbol, broken line) from a previous study compared to the same measure in mice infected with WtMu (black, solid symbol, solid line) or the autoluminescent strain (AlMu, red solid symbol, solid line) in the current study. In both experiments, all strains induced swelling in most mice within 4 to 5 weeks of infection. (TIF) [file pntd.0002598.s001.tif]

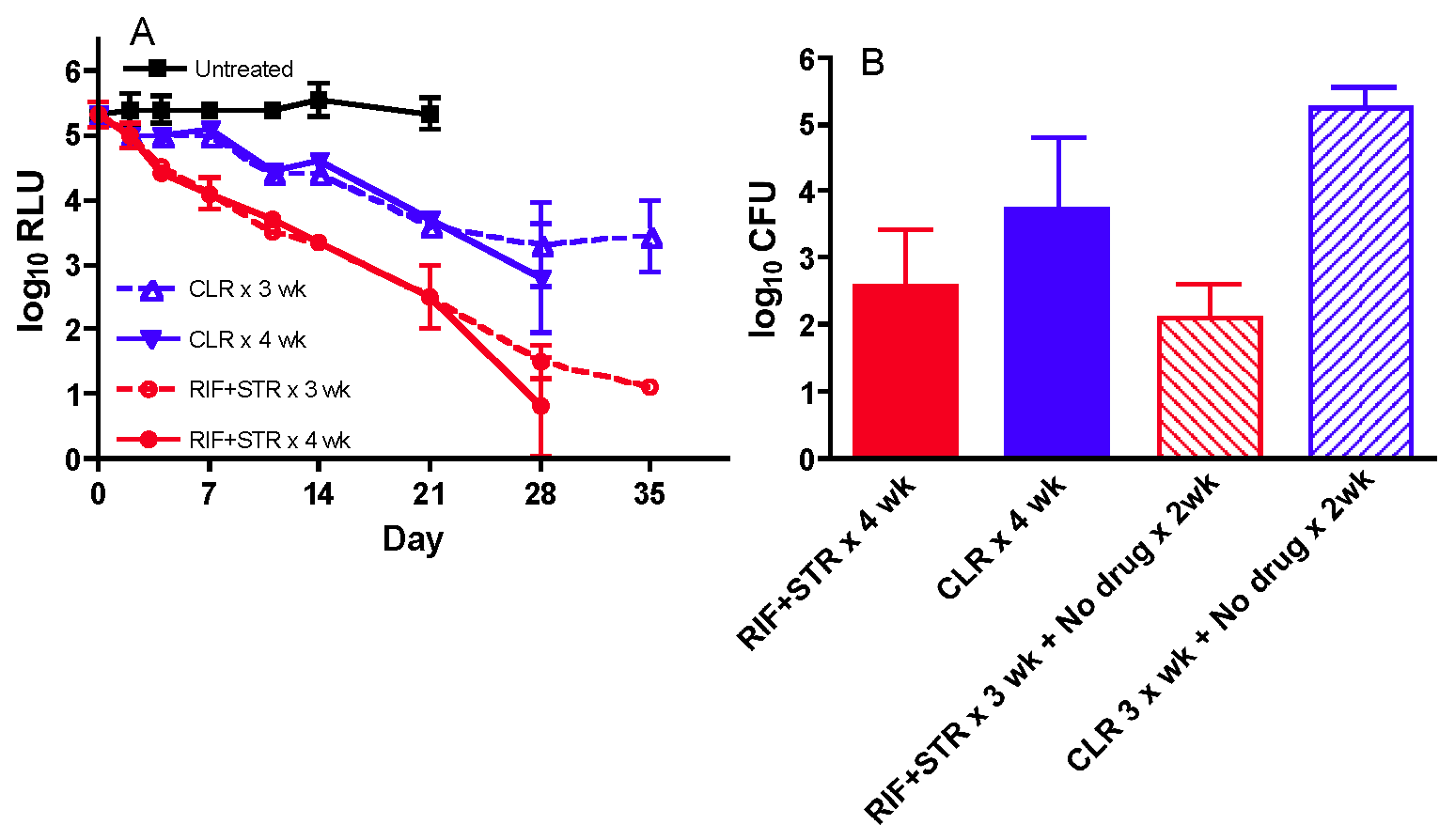

Supplement: Figure S2 — Post antibiotic effects of clarithromycin and combined rifampin and streptomycin. (A) Mice (N = 5 per group) with swollen footpads were treated for 3 weeks (broken lines) and had weekly RLU assessments for up to 5 weeks to determine the post antibiotic effects of clarithromycin (CLR, blue) monotherapy or rifampin and streptomycin (RIF+STR, red) combination therapy. Additional mice (N = 5 per group) were treated for 4 weeks and sacrificed at treatment completion (solid lines). (B) CFU counts in footpads of mice treated for 3 weeks and then assessed 2 weeks later (hatched bars) or treated for 4 weeks and then assessed at treatment completion (solid bars). (TIF) [file pntd.0002598.s002.tif]
